# Reversing immunosuppression in the tumor microenvironment of fibrolamellar carcinoma via PD-1 and IL-10 blockade

**DOI:** 10.1038/s41598-024-55593-6

**Published:** 2024-03-01

**Authors:** S. K. Daniel, K. M. Sullivan, L. K. Dickerson, R. J. E. van den Bijgaart, A. F. Utria, K. P. Labadie, H. L. Kenerson, X. Jiang, K. S. Smythe, J. S. Campbell, R. H. Pierce, T. S. Kim, K. J. Riehle, R. S. Yeung, J. A. Carter, K. C. Barry, V. G. Pillarisetty

**Affiliations:** 1 Department of Surgery, University of Washington School of Medicine, 1959 NE Pacific Street, Box 356410, Seattle, WA 98195 USA; 2 Public Health Sciences Division, Fred Hutchinson Cancer Research Center, Seattle, WA USA

**Keywords:** Cancer microenvironment, Cancer immunotherapy, Liver cancer

## Abstract

Fibrolamellar carcinoma (FLC) is a rare liver tumor driven by the DNAJ-PKAc fusion protein that affects healthy young patients. Little is known about the immune response to FLC, limiting rational design of immunotherapy. Multiplex immunohistochemistry and gene expression profiling were performed to characterize the FLC tumor immune microenvironment and adjacent non-tumor liver (NTL). Flow cytometry and T cell receptor (TCR) sequencing were performed to determine the phenotype of tumor-infiltrating immune cells and the extent of T cell clonal expansion. Fresh human FLC tumor slice cultures (TSCs) were treated with antibodies blocking programmed cell death protein-1 (PD-1) and interleukin-10 (IL-10), with results measured by cleaved caspase-3 immunohistochemistry. Immune cells were concentrated in fibrous stromal bands, rather than in the carcinoma cell compartment. In FLC, T cells demonstrated decreased activation and regulatory T cells in FLC had more frequent expression of PD-1 and CTLA-4 than in NTL. Furthermore, T cells had relatively low levels of clonal expansion despite high TCR conservation across individuals. Combination PD-1 and IL-10 blockade signficantly increased cell death in human FLC TSCs. Immunosuppresion in the FLC tumor microenvironment is characterized by T cell exclusion and exhaustion, which may be reversible with combination immunotherapy.

## Introduction

Fibrolamellar carcinoma (FLC) is a rare form of liver cancer that primarily occurs in otherwise healthy adolescents and young adults^1,2^. Patients with FLC often present with late stage disease that is not amenable to curative resection and rarely responds to chemotherapy^3,4^. Consequently, five-year recurrence free survival (RFS) for patients with FLC is lower than for those with hepatocellular carcinoma (HCC) despite a significantly healthier patient population^5–7^. The vast majority of FLC tumors harbor a characteristic chromosome 19 somatic microdeletion that creates a conserved fusion gene between part of the chaperone DnaJ heat shock protein 40 member B1 (*DNAJB1*) and the catalytic subunit of protein kinase A (*PRKACA*)^8–10^. The resultant DNAJ-PKAc fusion protein, but not PRKACA over-expression, is sufficient to induce tumors that resemble FLC in mice and is thought to play an important role in human FLC oncogenesis, potentially through recruitment of heat shock protein 70 and subsequent activation of ERK signaling^11^.

Immunotherapy for primary liver tumors has recently shown great promise, with HCC demonstrating a 20% durable response rate with programed cell death protein 1 (PD-1) blockade and improved RFS following adjuvant treatment with programed death-ligand 1 (PD-L1) and vascular endothelial growth factor (VEGF) inhibitors^12–14^ Relatively little is known about immune checkpoint blockade in FLC, and limited early stage trials show mixed results in a small number of patients^15–19^. Pre-clinical evidence suggests that FLC may ultimately be vulnerable to immunotherapy, as the FLC tumor microenvironment (TME) is characterized by high levels of immune checkpoint expression consistent with local immunosuppression and the DNAJB1-PRKACA fusion serving as a source of immunogenic neoantigens^20,21^. There is therefore an urgent need to better understand the mechanisms of immune resistance in the FLC TME and identify combination immunotherapeutic strategies that can specifically reverse this immunosuppression.

In this study, we used a combination of multiplex immunohistochemistry (mIHC) and flow cytometry to examine the effects of local immunosuppression within the FLC tumor microenvironment. Our results demonstrated that T cells are spatially excluded from the carcinoma compartment, with the highest density of CD8^+^ cytotoxic and CD4^+^ helper T cells found in adjacent non-tumor liver (NTL) and, intra-tumoraly, within dense fibrous stromal bands sequestered from tumor cells. Tumor-infiltrating lymphocytes (TILs) less frequently had expression of T cell activation markers but did have upregulation of exhaustion markers relative to those T cells in the adjacent NTL. Targeted T cell receptor (TCR) sequencing further revealed that TILs were rarely clonally expanded despite having high levels of TCR sequence conservation among individual patients, potentially representing the effects of shared DNAJB1-PRKACA neoantigens in an immunosuppressive microenvironment. Finally, we utilized a human tumor slice culture (TSC) experimental model system for the first time in FLC, allowing us to study the FLC TME of surgically resected tumor samples to demonstrate that a combination immunotherapy approach can successfully increase intra-tumoral cell death.

## Materials & methods

### Multiplex immunohistochemistry (mIHC) and image analysis

Formalin-fixed, paraffin embedded (FFPE) tissues from 6 patients (Table 1, patients 5–10) were sectioned at 4 microns onto slides and baked. Slides were stained on a Leica BOND Rx auto-stainer (Leica, Buffalo Grove, IL) using the Akoya Opal mIHC assay (Akoya Biosciences, Menlo Park, CA) as recommended by the manufacturer with the following changes: additional high stringency washes were performed after the secondary antibody and Opal fluor applications used high-salt TBST (0.05 M Tris, 0.3 M NaCl, and 0.1% Tween-20, pH 7.2–7.6). TCT was used as the blocking buffer (0.05 M Tris, 0.15 M NaCl, 0.25% Casein, 0.1% Tween 20, pH 7.6 + /– 0.1). Slides were incubated with primary antibodies for 1 h at room temperature, followed by OPAL Polymer HRP Mouse plus Rabbit (Akoya Biosciences) secondary antibody (Suppl. Table S1).

**Table 1:** Clinical characteristics.

Patient number	1	2	3	4	5	6	7	8	9	10	11	12	13	14
Sex	M	M	F	M	M	F	M	M	F	F	F	M	M	M
Age at diagnosis (yrs)	32	23	37	21	14	15	13	37	17	14	32	20	25	26
Tissue source	Hepatic primary	Hepatic primary	Hepatic primary	Peritoneal metastasis	Hepatic primary, retroperitoneal lymph node metastasis	Hepatic primary	Hepatic primary, retroperitoneal lymph node metastasis	Hepatic recurrence	Hepatic primary, peritoneal metastases	Hepatic primary	Hepatic primary	Hepatic primary	Hepatic primary	Hepatic primary
Age at surgery for tissue source	32	23	37	27	14, 15	16	13, 16	63	18,21	14	32	20	26	26
Neoadjuvant therapy	None	None	None	Gemcitabine, oxaloplatin, and bevacizumab	None	Sorafenib, chemo-embolization with mitomycin, cisplatin, and adriamycin	None	None	None	None	None	None	None	None
Initial resection tumor grade	2	2	3	N/A	N/A	N/A	2	N/A	2	2	2	2	2	N/A
Initial resection tumor size (cm)	5.5	10.5	8.0	11.0	11.0	20.0	9.5	13.0	9.5	10.5	5.0	12.0	14.7	11.0
Initial resection lymph nodes positive/total lymph nodes	N/A	5/9	26/35	N/A	2/2	0/8	0/2	0/2	0/8	0/3	0/5	0/9	0/9	3/21
Initial resection smallest margin (cm)	2.0	0.1	3.5	0.7	0.4	Transplant	0.2	Unknown	Positive	1.1	2.0	0.5	0.1	0.1
Adjuvant therapy	Additional resection, radiation, kinase inhibitor clinical trial	Gemcitabine and oxaloplatin, everolimus clinical trial, IFN-α and capecitabine	Additional resection and HIPEC, nivolumab	Additional resection × 7, panitimumab, erlotinib, IFN-α and capecitabine, radiation, lapitinib	Additional resection × 2, Sorafenib, Gemcitabine and Oxaloplatin	Liver transplant with initial resection, Cisplatin and Doxorubicin	Additional resection × 3, radiation	Additional resection × 3, RFA ablation, Cisplation and 5-FU, FOLFOX, radiation	Additional resection, DNAK-PKAc fusion peptide vaccine with Nivolumab and Ipilimumab, Lenvatinib	None	None	None	None	None
Days from initial resection to recurrence	508	176	404	300	59	N/A	669	8401	1346	N/A	N/A	N/A	384	N/A
Recurrence location	Liver, diaphragm	Liver, lung	Liver, peritoneum	Liver, retroperitoneal and mediastinal lymph nodes, peritoneum	Lung, mediastinum	None	Retroperitoneal lymph nodes, lung	Liver, mediastinal lymph nodes, chest wall	Pelvis, peritoneum	N/A	N/A	N/A	Liver	N/A
Days from initial resection until death or last clinical encounter (*currently alive)	1013	671	1345	2248	669	4691*	3852*	943	1840*	1389*	445*	48*	392*	68*

*HIPEC* heated intraperitoneal chemotherapy, *RFA* radiofrequency ablation.

*Currently alive on last clinical encounter through 12/31/2022.

Slides were mounted with ProLong Gold (Invitrogen/Life Technologies, Grand Island, NY) and cured for 24 h at room temperature in the dark before image acquisition at 20 × magnification on the Akoya Vectra 3.0 Automated Imaging System. Vectra images were spectrally unmixed using Akoya Phenoptics inForm software and exported as multi-image TIFFs representing a minimum of 15 mm^2^ tissue for use in HALO image analysis software (Indica Labs, Corrales, NM). Antibody positivity thresholds were calibrated for each individual slide, and cells were counted using automated nuclear detection. NTL, interface between tumor and NTL, and tumor, as well as intratumoral stroma and carcinoma compartment designations, were annotated and independently confirmed by an anatomic pathologist (RHP) using autofluorescence-generated false hematoxylin and eosin (H&E) images.

### RNA extraction and analysis

RNA was extracted from frozen paired FLC and NTL (Table 1, Patients 1–4) using the Qiagen (Hilden, Germany) RNEasy extraction kit per the manufacturer’s protocol. Samples were submitted for transcriptional analysis using NanoString Technologies (Seattle, WA) PanCancer Immune Profiling Panel^22^. Data were normalized and analyzed using NanoString Advanced Analysis software. Tumor and NTL RNA from patients with HCC and underlying cirrhosis from non-alcoholic steatohepatitis or hepatitis C virus were extracted and pooled before they were analyzed using the same protocols.

### Flow cytometry

Freshly resected FLC and NTL samples were immediately placed in phosphate buffered saline (PBS) in a sterile container and placed in a box of wet ice for transport to the laboratory. Both FLC and NTL were vigorously minced with surgical scissors and transferred to a GentleMACS C Tube (Miltenyi Biotec, San Jose, CA) with 20 µg/mL Liberase TL (Roche, Basel, Switzerland) and 10 µg/mL Dnase I (Roche) in 3 mL RPMI-1640 per gram of tissue. GentleMACS C tubes were incubated in the GentleMACS octo dissociator with heaters (Miltenyi Biotec), following the manufacturers dissociation protocol (37C_h_TDK_3, Miltenyi Biotec). Samples were then quenched with 7 mL FACS buffer (DPBS containing 3% fetal calf serum (FCS) and 1 mM EDTA), filtered through a 100 µm filter filter, and cells were collected by centrifugation (300xg, 10 min). Red blood cell lysis was performed with RBC lysis buffer (eBiosciences, Santa Clara, CA). After RBC lysis, cells were spun down and counted using the Vi-CELL BLU analyzer (Beckman Coulter, San Jose, CA). 1 × 10^6^ viable cells were used for flow cytometry as described below.

After single cell suspensions were made, cells were incubated with Fc receptor–blocking solution (Human TruStain FcX, BioLegend, San Diego, CA) to prevent non-specific antibody binding and Fixable Blue Dead Cell Stain (Invitrogen, Waltham, MA) for 15 min at room temperature in the dark. Cells were then washed with PBS and incubated with cell surface antibody mix diluted in FACS buffer containing BV stain buffer (BD biosciences, San Jose, CA) following manufacturer instructions for 20 min at room temperature in the dark. Cells were washed twice with FACS buffer and subsequently fixed in either Fixation Buffer (BD Biosciences) or in Foxp3/Transcription Factor Staining Buffer Set (eBioscience) if intracellular staining was required. See Suppl Table S2 for antibodies used in this study. After acquisition, data was exported in FCS 3.0 format and analyzed using FlowJo (Treestar, Ashland, OR) software. Samples were analyzed using a combination of manual gating and computational analysis approaches. Specifically, viable CD45^+^ events were exported as compensated data, and FLC and matched NTL were concatenated. Exported .fcs files were imported into the R environment utilizing the flowCore package^23^. Subsequently, we applied Arcsinh transformation with a scaling factor of 150. The data was down-sampled to 100,000 events, which were subsequently employed for dimensionality reduction through the Uniform Manifold Approximation and Projection (UMAP) algorithm^24^. Furthermore, we conducted Phenograph clustering (utilizing Rphenograph, with number of nearest neighbors (k) = 30)^25^. The resulting data were visualized in FlowJo software, UMAPs were overlayed with manually defined gates, enabling the identification of various immune cell subsets.

### TCR sequencing

FFPE curls (100 µm) of FLC and NTL were sent for DNA extraction and sequencing of the CDR3 T cell receptor (TCR) beta (β) chain by Adaptive Biotechnologies (Seattle, WA) using the ImmunoSeq Assay platform (Table 1, Patients 2, 5–10)^26^. Previously published TCR sequences for Merkel cell carcinoma (MC), non-small cell lung adenocarcinoma (LA), melanoma (ML), pancreatic ductal adenocarcinoma (PDA), colorectal adenocarcinoma (CRA), breast carcinoma (BC), and ovarian carcinoma (OC) were obtained through the immuneACCESS database^27–33^. Clonotypes were defined by productive complementarity-determining region 3ß (CDR3ß) amino acid sequence. Clonality, in bits, was calculated as:

$$Clonality=1+ \frac{\sum_{i=1}^{n}{{p}_{i}log}_{2}({p}_{i})}{{log}_{2}(n)} ,$$

where *n* is the number of unique clonotypes and ${p}_{i}$ is the probability of observing the *i*th clone. The pairwise overlap between repertoires *X* and *Y* was calculated using Morisita’s overlap index (MOI):

$$MOI=\frac{2\sum_{i=1}^{n}{x}_{i}{y}_{i}}{\left({D}_{X}+{D}_{Y}\right){N}_{X}{N}_{Y}},$$

where ${x}_{i}$ and ${y}_{i}$ are the number of times the *i*th clone is observed in each repertoire, ${N}_{X/Y}$ are the total number of unique clonotypes in each sample, and ${D}_{X/Y}$ is the Simpsons index for each sample. The Simpsons index is given as $\sum_{i=1}^{n}{p}_{i}^{2}$.

### Tumor slice culture

Fresh, sterile six mm FLC cores (Table 1, Patients 10–14) were acquired in the operating room^34,35^. Using a vibratome (Leica Biosystems, Wetzlar, Germany), the cores were sliced into 250 μm thick slices and placed on a PTFE membrane (EMD Millipore, Burlington, MA) with RPMI-based complete culture media in a 37 °C CO_2_ incubator as previously described^39^. The following day, slices were treated with either IgG1 isotype control monoclonal antibody (mAb) (BD Biosciences 553,447, 20 μg/mL), anti-PD1 mAb (BD Biosciences 562,138, 20 μg/mL), anti-IL10 mAb (BioLegend 501,407, 20 μg/mL), or anti-PD-1 mAb (20 μg/mL) plus anti-IL10 mAb (20 μg/mL). Slices were cultured for six days before fixation in formalin. Media were replaced at day 3 under the same treatment conditions.

### IHC

Single-color IHC was performed on FFPE tumor slices after six days of treatment as previously described using cleaved Caspase-3 (cC3) primary antibody (Cell Signaling Technologies, Danvers, MA, 1:200)^35^. Whole slide imaging and digital archiving was performed using the Nanozoomer Digital Pathology system (Hamamatsu, Shizuoka, Japan), and 20 × fields (7–13 fields per group) were automatically counted using QuPath or manually counted using Fiji ImageJ Cell Counter^36^.

### Statistics

When data was normally distributed (RNA seq, flow cytometry, TCR sequencing), paired and unpaired t-tests as well as analysis of variance (ANOVA) were used as appropriate. When data was not normally distributed (mIHC and tumor slice culture data), non-parametric Mann–Whitney *U* test or Wilcoxon matched pairs signed rank test as well as Kruskall-Willis test were used as appropriate. Correlations between clonality and mIHC data were made using linear regression. Graphs were created using PRISM (GraphPad, Boston, MA). The data generated in this study are available upon request from the corresponding author.

### Ethics approval and consent to participate

All investigations described in this article were conducted according to the principles expressed in the Declaration of Helsinki. De-identified clinical information and tissue were obtained in accordance with Institutional Review Board (IRB) protocols at the University of Washington (1852) and Seattle Children’s Hospital (SCH15277) and informed consent was obtained for all patients or their legal guardians. All research was performed in accordance with relevant guidelines/regulations of the University of Washington and Seattle Children’s Hospital IRB and all experimental protocol/s was/were approved by the University of Washington and Seattle Children’s Hospital IRB.

## Results

### T cells and macrophages infiltrate the FLC tumor microenvironment

**Figure 1:**
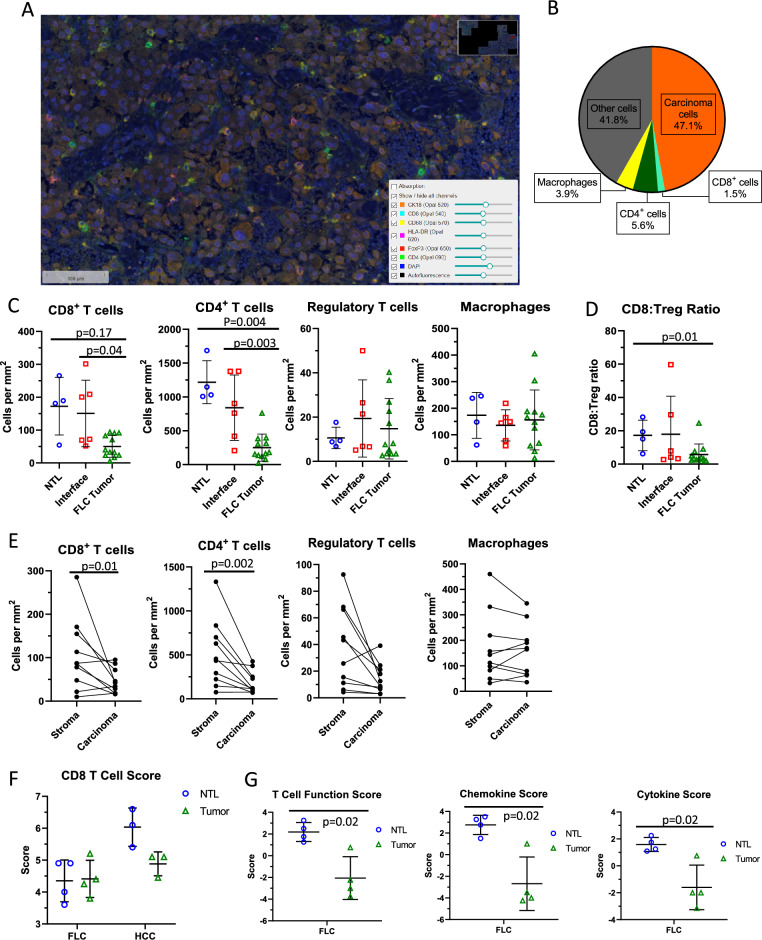
Using multiplex immunohistochemistry and RNA expression to define the immune landscape of FLC. (**A**) Representative mIHC 20 × field. (**B**) Overall average percentage of cell types in the FLC tumor compartment. (**C**) CD8 + T cell, CD4 + T cell, Regulatory T cell, and macrophage densities in adjacent NTL, interface, and tumor compartments in FLC, Mann–Whitney *U* test. (**D**) Ratio of CD8 + T cell: Regulatory T cell densities in adjacent NTL, interface, and tumor compartments in FLC, Mann–Whitney *U* test. (**E**) Paired CD8 + T cell, CD4 + T cell, Regulatory T cell, and macrophage densities in the stroma and carcinoma compartments of FLC tumor samples (5 patients, 9 slides), Wilcoxon matched-pairs signed rank test. (**F**) NanoString RNA expression CD8 T cell score comparing FLC NTL and tumor compartments to HCC NTL and tumor compartments, Mann–Whitney *U* test (**G**) NanoString RNA expression T cell function, chemokine, and cytokine scores of FLC NTL vs. tumor, Mann–Whitney *U* test.

In order to understand the immunosuppressive effects of the FLC TME, we first used mIHC to examine the prevalence and distribution of immune cells in the FLC TME (Table 1, patients 5–10; Fig. 1A). In the tumor compartment, we found that carcinoma cells comprised approximately 50% of all cells, with CD8^+^ T cells representing 1.5%, CD4^+^ T cells 5.6%, and macrophages 3.9% (Fig. 1B). Overall, we observed a CD4^+^:CD8^+^ ratio of 3.7:1.0, with regulatory T cells (Tregs; CD4^+^FOXP3^+^) accounting for 8.2% of CD4^+^ cells.

To quantify the spatial distribution of these immune cell types, we divided the tumor compartment into NTL, tumor interface, and intra-tumoral regions. We observed significantly more CD8^+^ T cells per mm^2^ in the tumor-NTL interface compartment compared to the tumor itself (p = 0.04), and a trend toward increased CD8^+^ T cell density in NTL compared to the tumor (Fig. 1C). CD4^+^ T cell density was significantly higher in the NTL and interface compartments than in the tumor (p = 0.004 and p = 0.003, respectively). No significant difference was found in Treg or macrophage density in the tumor compared to the NTL or interface (Fig. 1C). However, the ratio of CD8^+^ T cells to Tregs was significantly lower in the tumor compared to NTL (p = 0.01) and trended towards being lower in tumor compared to the interface (p = 0.15, Fig. 1D). Overall, these findings suggest that T cells in the FLC TME are physically excluded from the tumor compartment and may be subject to suppressive signaling.

We next examined the spatial distribution of immune cells within FLC tumors histologically, dividing the intra-tumoral region into nests of carcinoma cells intermixed with fibrous stromal bands. Similar mIHC examination demonstrated that the stromal compartment had significantly higher densities of CD8^+^ T cells (p = 0.01) and CD4^+^ T cells (p = 0.002). Macrophage and Treg density was similar between compartments (Fig. 1E). These data suggest that T cell exclusion from the carcinoma cells in FLC is accomplished by sequestration of tumor infiltrating lymphocytes in the fibrous stromal bands away from tumor cells.

### Lymphocyte populations within FLC have suppressed cytotoxic gene expression

Having defined the spatial distribution of immune cells in FLC, we used gene expression profiling of FLC and NTL to infer subtype and activation states of TILs (patients 1–4 in Table 1). We compared these data to those generated from classical HCCs and matched NTL. Using NanoString proprietary scores based on RNA expression, we found no significant difference in CD8^+^ T cell number between FLC and paired NTL, nor between FLC and HCC (Fig. 1F)^37^. There were significantly lower T cell function, cytokine, and chemokine scores in FLC compared with NTL (Fig. 1G), which suggests less cytotoxic activity. Similar trends were observed when evaluating individual gene expression for cell lineage markers as well as pro-inflammatory cytokines such as interferon gamma (IFN-γ) and tumor necrosis factor (TNF) (Suppl. Fig. S1A, S2E). These data suggest that T cells in FLC are either naïve or functionally suppressed.

Surprisingly, few genes in this dataset were upregulated in FLC (Suppl Fig. S1F). Arginase 2 (*ARG2*), a mitochondrial enzyme involved in the urea cycle, was induced in FLC compared to paired NTL (p = 0.007) and HCC (p = 0.02). Decreased L-arginine levels have been shown to impair CD8^+^ T cell cytotoxic function^38^. Both *IL32* and *IL34* were more highly expressed in FLC than NTL (p = 0.02 and p = 0.006, respectively), and *IL34* was also more highly expressed in FLC compared to HCC (p = 0.005). While less is known about IL-34, except that it can increase monocyte activity, IL-32 has been implicated in fibrosis and cancers associated with chronic inflammation, including HCC^39,40^. Platelet-derived growth factor receptor β (*PDGFRB*) gene expression was significantly higher in FLC compared to paired NTL (p = 0.01) and HCC (p = 0.04). In the liver, the *PDGFRB* is highly expressed by fibroblasts and smooth muscle cells, and its expression increases with inflammation and fibrogenesis^41^. Similar inductions of *ARG2*, *IL34*, and *PDGFRB* expression were not seen in HCC compared to paired NTL, suggesting this pattern is unique to FLC, although exact mechanisms are unclear.

### Immune profiles in the tumor microenvironment of FLC

**Figure 2:**
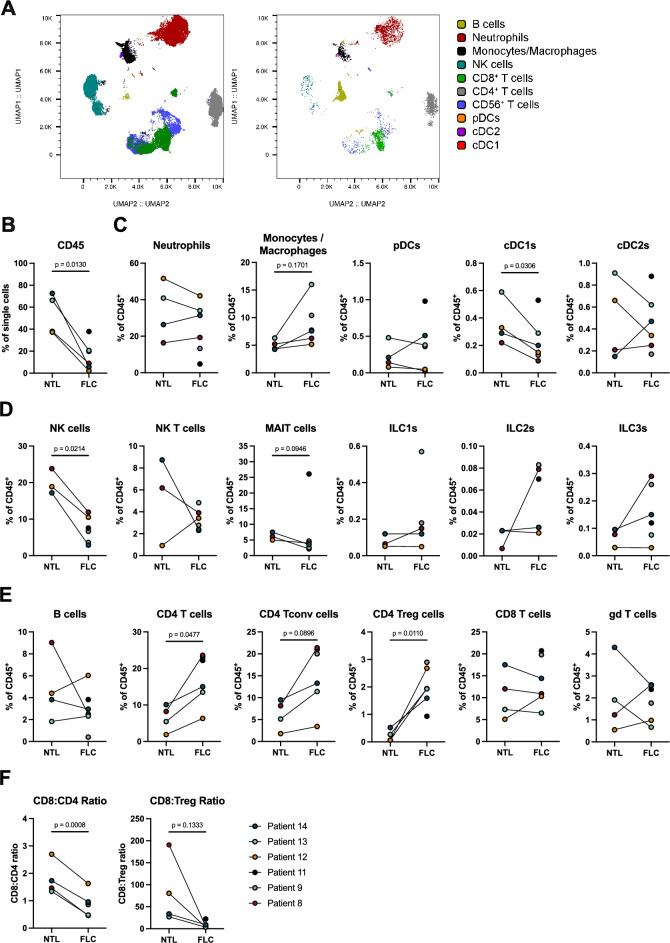
Immune profiles in the tumor microenvironment of fibrolamellar carcinoma and NTL. (**A**) UMAP displaying myeloid-centered panel flow cytometry analysis of live CD45^+^ cells from NTL (left) and FLC (right) of patient 14. Each color represents a different immune population. (**B**) Frequency of CD45^+^ among live cells in tumor and NTL. (**C**) Frequency of myeloid populations among CD45^+^ cells in tumor and NTL. (**D**) Frequency of innate lymphocyte populations among CD45^+^ cells in tumor and NTL. (**E**) Frequency of lymphocyte populations among CD45^+^ cells in tumor and NTL. (**F**) Ratio of CD8^+^ cells to CD4^+^ and Treg cells in tumor and NTL; All paired *T*-test, parametric.

To further understand the constituent immune cells in the FLC TME, we catalogued the immune landscape in six FLC samples and four paired NTL with flow cytometry using myeloid, innate(-like) lymphocyte and T cell-centered panels. The myeloid-centered panel distinguishes all canonical dendritic cell (DC) subsets, with parallel enumeration of monocytes/macrophages, T cells, B cells and natural killer (NK) cells^42,43^. UMAP analysis of the entire live immune compartment stained with the myeloid-centered panel revealed the presence of diverse immune cell populations, which were separated clearly (Fig. 2A) Neutrophils and T cells were found to be the most common populations of the tumor-infiltrating immune cells, both in FLC and NTL (Fig. 2A).

Flow cytometric analysis of FLC showed fewer CD45^+^ immune cells compared to matched NTL (Fig. 2B). Generally, we found large differences in both the variety and composition of myeloid immune cells (Fig. 2C), innate(-like) lymphocytes (Fig. 2D), and lymphocytes (Fig. 2E) across patients (gating strategies outlined in Suppl. Fig. S2). Regarding myeloid cell population abundance, a trend towards increased monocyte/macrophage frequency was observed in FLC compared to NTL (Fig. 2C). Conventional type 1 DCs ( cDC1s), which have a known role in initiating T cell responses to cancer, were found to be significantly lower in number in FLC compared to NTL, while no differences were observed in the frequencies of neutrophils, plasmatocytoid DCs (pDCs) and conventional type 2 DCs (cDC2s, Fig. 2C)^44–46^. Regarding innate-like lymphocytes, we observed a significant reduction in the proportion of NK cells and a slight decrease in mucosal-associated invariant T (MAIT) cells in FLC compared to NTL (Fig. 2D); however, there was no difference in the abundance of other innate cell populations such as NKT cells, innate lymphoid cells type 1 (ILC1s), ILC2s and ILC3s observed between FLC and NTL (Fig. 2D, Suppl. Fig S3)^47,48^.

In FLC samples, a significantly higher frequency of CD4^+^ T cells among CD45^+^ immune cells was observed compared to NTL (Fig. 2E). This finding was consistent with our mIHC analysis in FLC, in which a higher absolute number of CD4^+^ T cells per mm^2^ was observed compared to other immune cell types. CD4^+^ Tregs (p = 0.011) were found specifically to be higher in FLC, while there was only a trend towards increased conventional CD4^+^ T cells (p = 0.0896) in FLC (Fig. 2E). No differences were observed in the proportions of CD8^+^ T cells, gamma-delta (γδ) T cells and B cells between FLC and NTL (Fig. 2E). The increase in CD4^+^ T cells in FLC resulted in a significant decrease in the CD8^+^ to CD4^+^ T cell ratio in FLC compared to NTL (Fig. 2F). Furthermore, we found a decreased ratio of CD8^+^ T cells to Tregs in FLC compared to NTL (Fig. 2F). The increased prevalence of CD4^+^ T cells over CD8^+^ T cells and decreased CD8^+^ T cell to Treg ratio in FLC compared to NTL is consistent with our spatial mIHC analysis (Fig. 1B). Taken together, these results suggest an immune excluded and immunosuppressive microenvironment in FLC, with a TME that is biased towards CD4^+^ T cells, and contains fewer cDC1s and NK cells.

### The T cell infiltrate in FLC is skewed toward an effector memory phenotype that expresses PD-1 and CTLA-4

**Figure 3:**
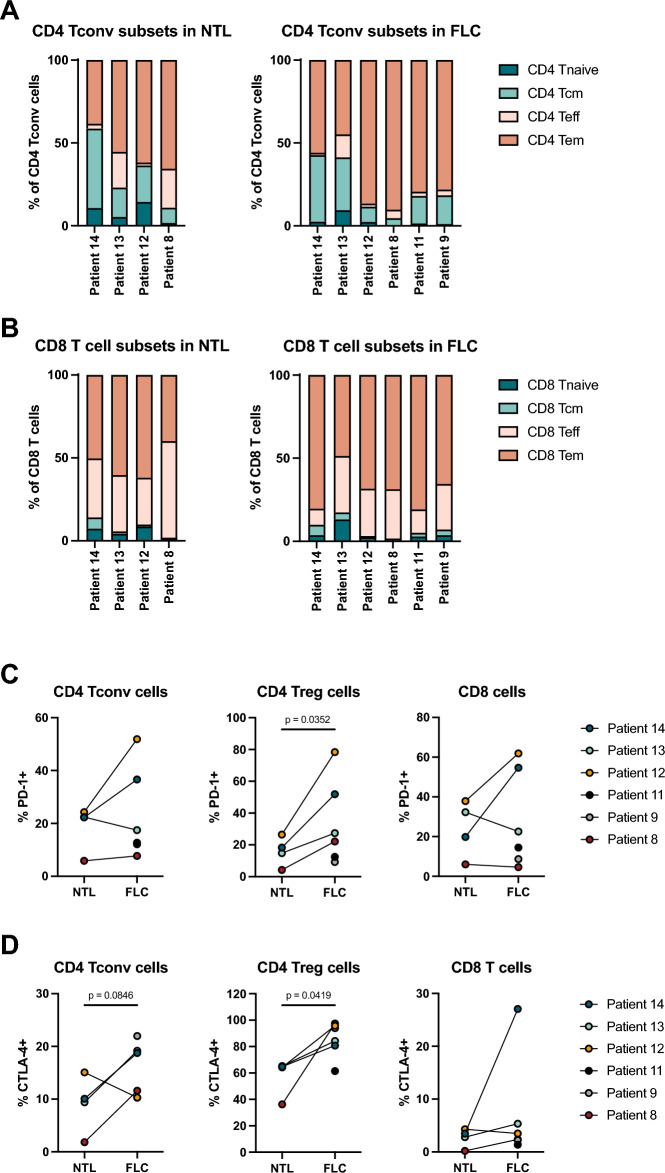
Characterization of T cell subsets and their PD-1 and CTLA-4 expression in FLC and NTL. (**A**) CD4 T cell subsets in NTL (left) and FLC (right). (Tnaive = CD45RA^+^CCR7^+^, Tcm = CD45RA^-^CCR7^+^, Teff = CD45RA^+^, CCR7^-^, Tem = CD45RA^-^CCR7^-^) (**B**) CD8 T cell subsets in NTL (left) and FLC (right). (**C**) and (**D**) PD-1 and CTLA-4 expression by conventional CD4 T cells, regulatory CD4 T cells and CD8 T cells in NTL and FLC; All paired *T* test, parametric.

We next characterized the phenotype of tumor-infiltrating CD4^+^ and CD8^+^ T cells using flow cytometry. We included C–C chemokine receptor type 7 (CCR7) and CD45RA to define naïve (Tnaïve), central memory (Tcm), effector memory (Tem) and terminally differentiated T effector (Teff) cells in FLC and NTL samples (Suppl. Fig. S2). Both the CD4^+^ and CD8^+^ T cells were predominantly Tem in FLC, and this proportion was increased in FLC compared to NTL for both cell types, suggesting that the majority of intratumoral T cells are antigen-experienced (Fig. 3A,B). The proportion of CD4^+^ and CD8^+^ Teff were decreased in FLC compared to NTL, while the proportion of Tcm and Tnaïve cells were unchanged.

Little information exists on the expression pattern of targetable immune checkpoint molecules on T cells in FLC^20^. Therefore, we analyzed the expression of PD-1 and cytotoxic T-lymphocyte-associated antigen 4 (CTLA-4) on T cells in FLC and NTL. We observed heterogenous expression of PD-1 and CTLA-4 on conventional CD4^+^ T cells, CD4^+^ Tregs and CD8^+^ T cells in FLC as well as NTL (Fig. 3C,D). CD4^+^ Tregs infiltrating FLC expressed significantly more PD-1 and CTLA-4 than Tregs present in NTL (Fig. 3C). In patients 12 and 14, we observed an increase in conventional CD4^+^ T cells and CD8^+^ T cells expressing PD-1 in FLC compared to NTL, while for patients 8 and 13 the PD-1 expression was comparable between FLC and NTL (Fig. 3C). In three of four patients, an increase in conventional CD4^+^ T cells expressing CTLA-4 was observed, while CTLA-4 was rarely detected on CD8^+^ T cells, except for CD8^+^ T cells infiltrating the FLC of patient 14 (Fig. 3D).

Together, these data demonstrate that the T cell infiltrate in FLC consists mainly of antigen-experienced effector memory CD8^+^ and CD4^+^ T cells, and that naïve T cells are largely absent. Furthermore, we found expression of immune checkpoint molecules in FLC, suggesting T cell exhaustion and the development of regulatory mechanisms that play a role in suppressing local antitumor immune function.

### FLC tumor infiltrating TCRs are relatively unexpanded yet highly conserved

**Figure 4:**
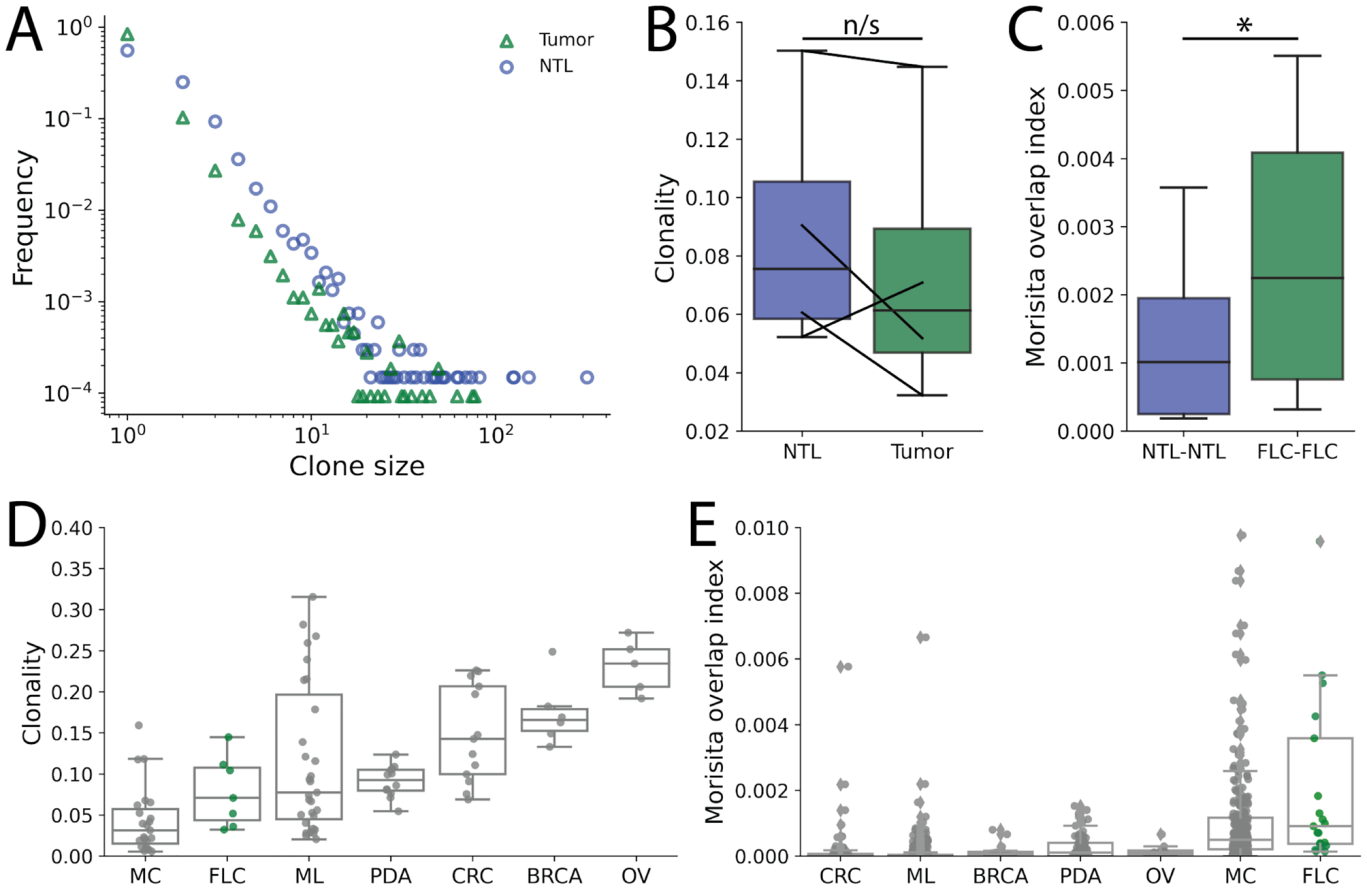
T cell receptor (TCR) sequencing demonstrates low clonality but increased repertoire conservation. (**A**) Clone size distribution for FLC primary tumor and NTL samples down sampled to 20,000 unique CDR3ß amino acid sequence clonotypes. NTL appear to be more frequently expanded, with 3 NTL clones seen more than 100 times. (**B**) Clonality, with lower values corresponding to more polyclonal samples, demonstrates that FLC primary tumor samples are less expanded in 3 of 4 paired samples. While NTL samples tended towards higher clonality, this effect was not significant in our limited sample size (n/s- not significant by paired *t* test). (**C**) Morisita’s overlap index (MOI), a measure of repertoire overlap with higher values directly corresponding to a larger proportion of shared sequences between two samples, was calculated in a pairwise fashion for all NTL or FLC samples. FLC primary tumors samples more often shared TCR clonotypes across individuals than NTL samples (*p* < 0.03 by paired t-test). (**D**) and (**E**) FLC primary tumor samples had the lowest clonality but highest MOI when compared with melanoma (ML), pancreatic adenocarcinoma (PDA), colorectal adenocarcinoma (CRC), breast carcinoma (BRCA), and ovarian carcinoma (OV) samples downloaded from the immuneACCESS database^27–33^. A similar pattern of low clonality but high overlap between individuals was also seen for Merkel cell carcinoma (MC).

Baseline intratumoral T cell receptor (TCR) clonality has previously been shown to positively correlate with prognosis and immunotherapy response across a variety of tumor types^49,50^. The DNAJ-PKAc fusion protein represents a potential foreign antigen and target for either TCR selection of different rearrangement events. However, the various observed immunosuppressive features within the FLC TME could limit clonal expansion of various TCRs, including those recognizing tumor neoantigens. To better understand the function of TILs in FLC patients, we first performed high-throughput sequencing of the TCRß chain from four FLC primary tumors with paired NTL samples (Table 1, patients 2, 8–10)^26,51^. Identifying between 5866 and 144,221 productive rearrangements per sample, we defined a TCR clonotype as the β chain complementarity-determining region 3 (CDR3β) amino acid sequence. We found that while clone sizes for both FLC and paired NTL followed a characteristic power-law distribution, TCRs in NTL samples appeared to have more frequently undergone clonal expansion than those clonotypes within the tumors (Fig. 4A).

To further quantify repertoire dynamics within FLC, we calculated the clonality of each sample as one plus the normalized Shannon entropy. In brief, clonality ranges between zero and one with a decreasing clonality score corresponding to increasing repertoire diversity (e.g. a clonality of one represents a repertoire composed of only a single clone and thereby minimum diversity). We found that the clonality of NTL samples was higher than their primary tumors in three out of four patients (Fig. 4B), though the observed increase in NTL repertoire clonality was not statistically significant with this limited sample size (p = 0.37). Furthermore, there was a strong inverse correlation between the clonality of FLC samples and the densities of CD8^+^, CD4^+^, and Tregs in the carcinoma compartment based on mIHC (Suppl. Fig. S4A). This finding clearly demonstrates that large clonal expansions do note explain the relatively high TIL frequencies in certain FLCs. Together, these findings are suggestive of an immunosuppressive TME that inhibits clonal expansion of TILs in FLC.

FLC is unique in that it arises in healthy liver parenchyma due to expression of the DNAJ-PKAc fusion protein^2^. We therefore theorized that this unique fusion protein could represent an immunogenic neoantigen shared across individuals, which could in turn generate shared TCR responses despite the lack of large clonal expansions. To test this hypothesis, we calculated Morisita’s overlap index (MOI) to evaluate the pairwise proportion of rearrangements coding for the same CDR3β amino acid sequence in FLC. Here, a value of 0 indicates no shared sequences, while a value of 1 indicates that all sequences are shared between two samples. Calculating the MOI for all pairwise combinations of either NTL or FLC tumor samples, we indeed found that the tumor samples had higher rates of TCR sharing between individuals (Fig. 4C). Similar to previous findings in healthy peripheral blood samples, these shared TCR sequences typically had higher generation probabilities and were more frequently known to recognize common viral epitopes relative to sequences unique to a single individual (Suppl Fig. S4B)^52–54^. However, as these known TCR sequences are present in both FLC tumor and NTL samples, these observations do not explain the increased conservation of TCR sequences among FLC tumors, and none have been directly linked to the DNAJ-PKAc fusion protein.

To further delineate the importance of conserved TCR sequences across FLC, we downloaded TCRβ sequencing data from six other solid tumor types in the immuneAccess database^27–33^. Our seven FLC primary tumor samples (including three additional samples without paired NTLs) had a lower average clonality but significantly higher MOI than melanoma (ML), pancreatic ductal adenocarcinoma (PDA), colorectal adenocarcinoma (CRC), breast carcinoma (BRCA), and ovarian carcinoma (OV) (Fig. 4D,E). Merkel cell carcinomas (MC), the majority of which were positive for the common Merkel cell polyomavirus T-antigen, displayed a similar pattern of relatively low clonality but high repertoire overlap across individuals. Together, these results highlight a high degree of TCR sequence conservation across FLC tumors. These findings could be consistent with an enrichment of FLC TILs with shared TCRs recognizing common neoantigens despite the immunosuppressive TME limiting the expansion and in vivo efficacy of these tumor-specific clonotypes. We thus hypothesized that reversing the suppressive TME in FLC could enable these putatively neoantigen specific TILs to effectively kill tumor cells.

### Blockade of PD-1 and IL-10 can promotes FLC cell death in human TSC

**Figure 5:**
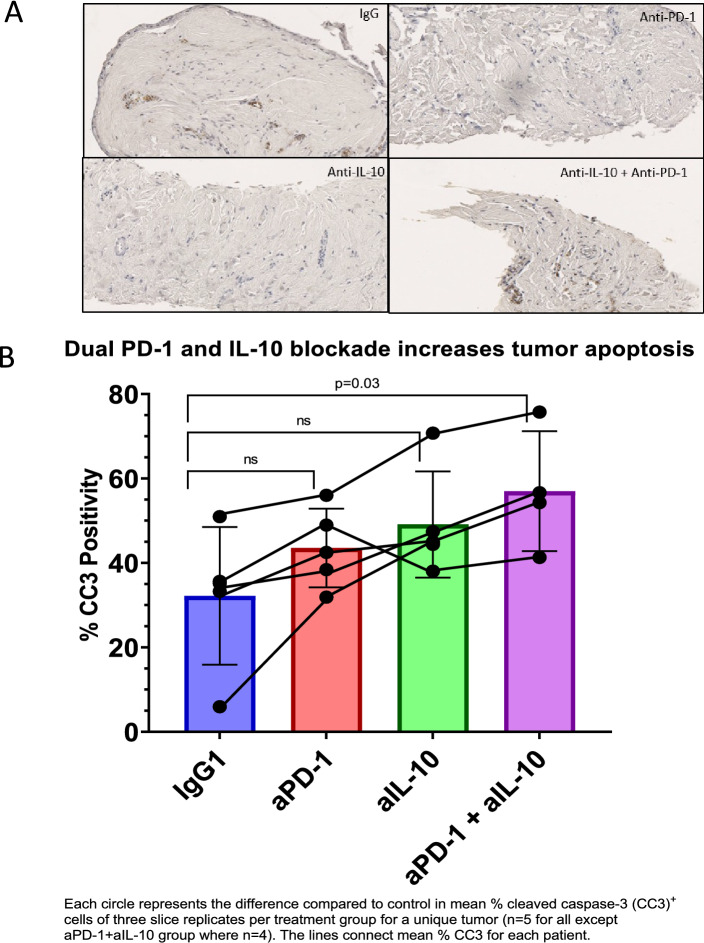
Tumor slice culture treatment with anti-PD-1 and anti-IL-10. (**A**) Representative 20 × fields of tumor slices from patient 10 stained for cleaved-Caspase-3 (cC3) after treatment with IgG mAb control, anti-PD-1 mAb, anti-IL-10 mAb, and anti-PD-1 mAb + anti-IL-10 mAb. (**B**) Average % cC3 raw positivity by treatment group for all patients combined. Each circle represents the difference compared to control in mean % cC3 + cells of three slice replicates per treatment group for a unique tumor (n = 5 for all except the aPD-1 and aIL-10 group where n = 4). The lines connect mean % cC3 + for each patient. Mann–Whitney *U* test.

Using ex vivo TSC from five patients, we tested the hypothesis that immunotherapy has the potential to rescue endogenous anti-tumor immune responses in FLC (Table 1, Patient 10–14)^34,35^. To augment the effectiveness of ICI in the immunosuppressive nature of the FLC TME, we decided to target macrophage pathways due to the trend towards increased in monocyte/macrophage presence in FLC compared to NTL. Due to our prior identification of CD8^+^ T cell-mediated tumor cell death after IL-10 blockade in colorectal liver metastases, we evaluated PD-1 and IL-10 blockade alone or in combination in tumor slices to target T cell and macrophage-mediated suppression, respectively^55^. FLC slice cultures were treated with IgG control mAb, anti-PD-1 mAb, anti-IL-10 mAb, or anti-PD-1 plus anti-IL-10 mAbs for 6 days and tumor cell apoptosis was measured using cleaved caspase 3 (cC3) IHC (Fig. 5A). The average percentage of tumor cells expressing cC3 was 32.2% (SD 16.3) for IgG, 43.5% (SD 9.3) for anti-PD-1, 49.1% (SD 12.5) for anti-IL-10, and 56.9% (SD 14.2) for anti-PD-1 and anti-IL-10 combination treatment. Notably, the combination of anti-PD-1 and anti-IL-10 induced significantly more tumor cell apoptosis than IgG control (p = 0.03), while the difference between either of the individual treatment groups compared to IgG control (p = 0.30 for anti-PD-1 and p = 0.09 for anti-IL-10) did not reach statistical significance (Fig. 5B). Generally, more intra-tumoral cell apoptosis was observed when FLC slice cultures were treated with anti-IL-10 mAb alone compared to anti-PD1 mAb alone, but TSCs from patient 11 were an outlier in that more cC3 positivity was observed in response to PD-1 blockade alone compared to IL-10 blockade alone (Suppl Fig. S5). Interestingly, that patient also had the lowest percentage Tregs. Conversely TSCs from patient 13, who had the highest percentage of monocytes/ macrophages in FLC by flow cytometry, had a large increase in apoptosis for anti-IL-10 alone compared to IgG control. Together, these results indicate that combination PD-1 and IL-10 blockade can lead to increased intra-tumoral cell death.

## Discussion

FLC has a unique TME, which presents challenges as well as opportunities in considering immunotherapy for these patients. Our multiplex IHC and Nanostring gene expression analyses demonstrated a lower number of both CD8^+^ and CD4^+^ T cells in FLCs than in NTL or the tumor-NTL interface but a relatively preserved number of Tregs in tumors. These findings are consistent with previously published data regarding the immune landscape of FLC^20^. Additionally, we found that CD8^+^ and CD4^+^ T cells located within the tumor compartment are cordoned off in stromal bands, and thus sequestered from carcinoma cells. Using flow cytometry, we observed within the immune cell population a skewing towards increased CD4^+^ T cells in FLC, both conventional and Tregs, and reduced NK cells and cDC1s in FLC compared to paired NTL. Both mIHC and flow cytometry demonstrated reduced CD8:Treg ratios in FLC compared to NTL, reinforcing the suppressive nature of the FLC TME. Interestingly, the number of naïve T cells in FLC is low, with Teff and Tem phenotypes comprising the majority of the CD8^+^ fraction, and Tem comprising the majority of CD4^+^ T cells. The significantly increased presence of CD4^+^ Tregs, especially those that are PD-1^+^, in FLC could contribute to the decreased clonality and spatial separation of effector T cells from the carcinoma cells. While the expression of the immune checkpoint molecules PD-1 and CTLA-4 was not significantly increased in CD8^+^ T cells in the FLC versus NTL, these markers were more frequently expressed on CD4^+^ Tregs in tumors. These findings suggest that targeting the large population of CD4^+^ T cells in FLC may be necessary to reverse immunosuppression in these tumors.

Once we discovered that effector T cells are present in FLC based on flow cytometry data, we further evaluated whether these T cells had undergone clonal expansion in response to tumor antigen exposure. We hypothesized that there would be a few shared dominant clones (i.e., that clonality would be high) due to the low percentage of naïve CD8^+^ T cells, but we actually found more clonal expansion in paired NTL than FLCs themselves. Additionally, clonality did not increase by lymphocyte cell density on mIHC suggesting that the immunosuppressive nature of the FLC TME is preventing clonal expansion. We next evaluated specific TCR sequences to determine whether there is overlap between FLCs from patients, all of whom express the shared DNAJ-PKAc fusion protein, despite the lower than expected clonal expansion. Compared to paired NTL, there were more shared TCR sequences between FLCs, suggesting that downstream effects of the DNAJ-PKAc fusion protein create similar neoepitopes that are recognized by T cells. A major source of neoepitopes for most tumor types is a result of a high tumor mutational burden (TMB), however, FLC is characterized by low TMB^56,57^. Therefore, we compared the clonality of FLC TILs to that of other tumor types. We found lower clonality but increased TCR sequence overlap in FLC compared to most other solid tumor types^27–33^. Interestingly, there was a similar pattern of low clonality and higher TCR overlap between patients in Merkel cell carcinoma, which also has a common driver (the polyoma virus) in most patients^27^. Taken together, these data suggests that although clonal expansion of T cells has not typically occured in the native FLC TME, it could be possible if the immunosuppressive TME was modulated.

Since immune checkpoint inhibitor (ICI) monotherapy has been largely ineffective for many tumor types, especially in the setting of a low TMB, we pursued a targeted combination blockade approach to modulating immunosuppression in FLC^58^. The immune tolerant nature of the liver is dependent on many immunosuppressive cytokines, including IL-10, which is produced by certain phenotypes of macrophages and Tregs^59–61^. IL-10 is known to enhance Treg function while causing effector CD4^+^ cell anergy^62^. Our group previously demonstrated that IL-10 blockade helps increase T-cell mediated tumor death in colorectal liver metastases dependent on both MHC class I and II antigen presentation^55^. With the increased CD4^+^ Tregs and effector CD4^+^ T cells in FLC tumor, we hypothesized that IL-10 blockade could work in FLC in conjunction with blockade of PD-1 to reactive effector CD8^+^ and CD4^+^ T cells^63^. We found that combination PD-1 and IL-10 blockade led to increased intra-tumoral cell apoptosis compared to IgG control after 6 days, while anti-PD-1 or anti-IL-10 monotherapy did not lead to significantly increased apoptosis. These data are limited by our small sample size (n = 5) necessitated by the extremely low incidence of FLC and responses were not uniform, with one patient not responding synergistically to the combined treatment. This patient who did not demonstrate a synergistic response was also an outlier in that their tumor contained the most CD45^+^ cells, increased proportions of DCs and MAIT cells, and decreased proportions of neutrophils and CD4^+^ Tregs. These altered cell populations may have resulted in decreased baseline IL-10 which would make its blockade less effective. Further work is being done to confirm the exact mechanism of cell death; however, we hypothesize that reactivation of sequestered effector CD8^+^ T cells through combination of PD-1 and IL-10 blockade is possible in FLC, likely via effects on APCs including CD4^+^ cells and macrophages.

The cellular metabolic state of FLC may also contribute to decreased intratumoral CD8^+^ T cell density and dysfunction. *ARG2*, one of the genes we found to be upregulated in FLC, could affect cell metabolism in both tumor and immune cells. Arginase catalyzes the conversion of arginine to ornithine and urea in the urea cycle, which influences the metabolic pathways that lead to production of pyrimidine, polyamines, and glutamine, all of which are used by tumor cells for rapid cell division^64,65^. Additionally, arginine deficiency prevents T cell proliferation and downregulates a signaling element on the TCR with subsequent T cell dysfunction^66^. Arginine levels have also shown to be correlated with ICI response in other cancers, and arginase 2 has been shown to downregulate macrophage-mediated inflammation via IL-10^67,68^. While the exact signaling mechanism downstream of the DNAJ-PKAc fusion kinase that affects metabolism is unknown, there is ongoing research in this area with respect to both targeted treatment and immunotherapy.

In summary, this study provides insights into the complex immune landscape of FLC. A major limitation of the study is the relatively small sample sizes available to study given the extreme rarity of this tumor type. Nonetheless, using multiple orthogonal approaches, we are able to glean new insights into the the immune TME of FLC and show that the immunosuppressive environment is potentially reversable. We propose that immunotherapy targeting specific immunosuppressive features of FLC is a worthy pursuit for patients with this devastating disease. Due to the lack of efficacy seen in single-agent ICI in many gastrointestinal tumors so far, we anticipate that combination therapy such as co-blockade of PD-1 and IL-10 will offer a higher chance of success^15^. Further work focusing on elucidating the interplay between immune, chemokine, stromal, and metabolic factors in the FLC TME that affect T cell infiltration, phenotype, and function may reveal additional targets for combination immunotherapy.

### Supplementary Information

Supplementary Information.

## Data Availability

All de-identified data will be available after publication upon request to the corresponding author. NanoString data are available through their data servers.

## Abbreviations

FLC: Fibrolamellar carcinoma
mIHC: Multiplex immunohistochemistry
TCR: T cell receptor
PD-1: Programmed cell death protein 1
IL-10: Interleukin 10
cC3: Cleaved caspase-3
IHC: Immunohistochemistry
NTL: Non-tumor liver
HCC: Hepatocellular carcinoma
DNAJ: Heat shock protein 40
PKAc: Catalytic subunit of protein kinase A
ICI: Immune checkpoint inhibitors
PD-L1: Programed death-ligand 1
TILs: Tumor-infiltrating lymphocytes
FFPE: Formalin-fixed, paraffin-embedded
IRB: Institutional review board
CC-IRB: Cancer consortium institutional review board
MOI: Morisita’s overlap index
PCA: Principal component analysis
SNN: Shared nearest neighbor
mAb: Monoclonal antibody
TME: Tumor microenvironment
HLA: Human leukocyte antigen
PDGFRB: Platelet-derived growth factor receptor β
MC: Merkel cell carcinoma
LA: Non-small cell lung adenocarcinoma
PDA: Pancreatic ductal adenocarcinoma
CRA: Colorectal adenocarcinoma
BC: Breast carcinoma
ML: Melanoma
OC: Ovarian carcinoma
scRNAseq: Single-cell RNA sequencing
TCGA: The Cancer Genome Atlas
LIHC: Liver hepatocellular carcinoma
RFS: Recurrence-free survival
TSC: Tumor slice cultures
IFN-γ: Interferon gamma
TNF: Tumor necrosis factor
UMAP: Uniform manifold approximation and projection
DC: Dendritic cell
NK cell: Natural killer cell
MAIT cell: Mucosal-associated invariant T cell
Treg: Regulatory T cell
ILC: Innate lymphoid cells
conventionalT cell: Conventional T cell
γδ T cell: Gamma-delta T cell
Tcm: Central memory T cell
Tem: Effector memory T cell
Teff: Effector T cell
CTLA4: Cytotoxic T-lymphocyte-associated antigen 4
TMB: Tumor mutational burden
