# Supplementary material for: Reversing immunosuppression in the tumor microenvironment of fibrolamellar carcinoma via PD-1 and IL-10 blockade
Source: Sci Rep. 2024 Mar 1;14:5109. doi: 10.1038/s41598-024-55593-6 (PMC10907637; doi:10.1038/s41598-024-55593-6)
Supplement: Supplementary file 1 — Supplementary Information. [file 41598_2024_55593_MOESM1_ESM.pdf]

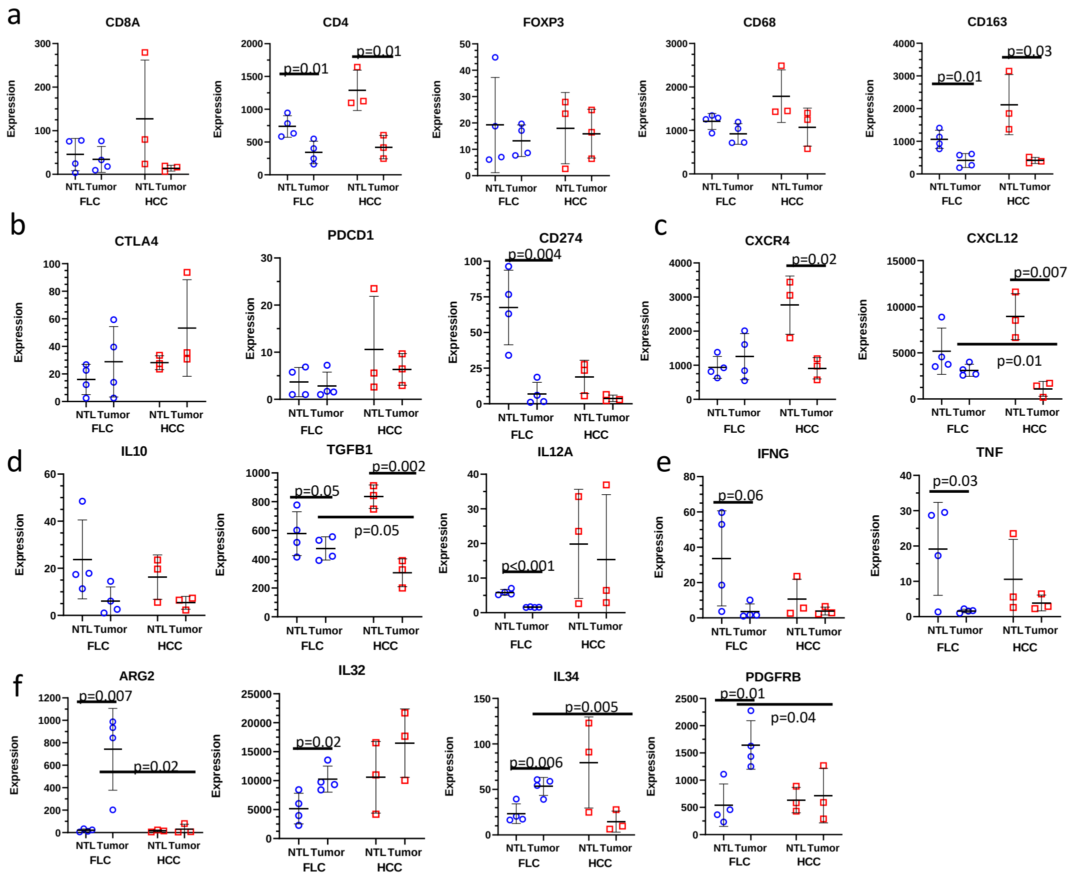

Supplementary figure S1.

Individual RNA expression counts for (a) immune cell type markers, (b) checkpoint inhibition markers, (c) chemokine and chemokine receptors, (d) anti-inflammatory cytokines, (e) pro-inflammatory cytokines, and (f) genes upregulated in FLC tumor

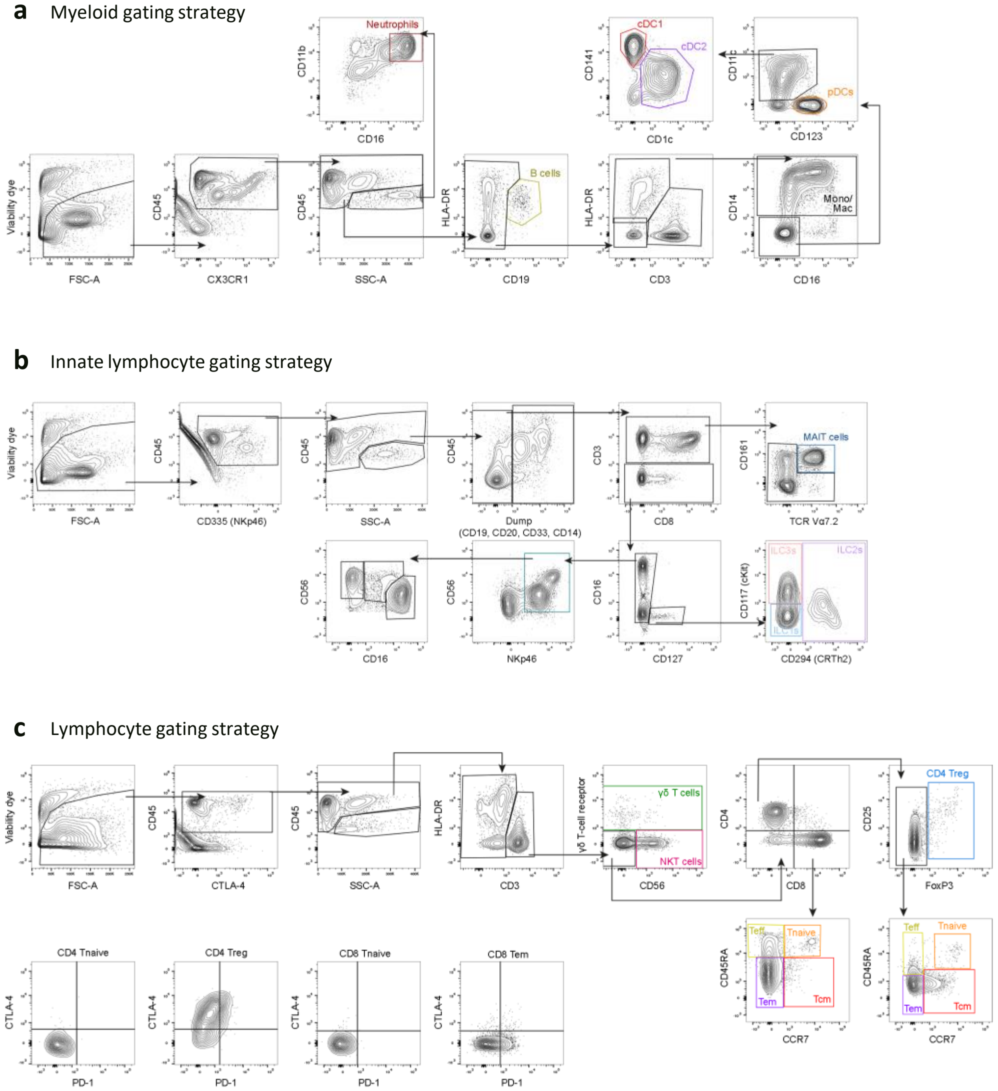

Supplementary figure S2.

Representative flow cytometric gating strategy to define tumor-infiltrating myeloid (a), innate lymphocytes (b) and lymphocyte (c) populations in FLC and NTL.

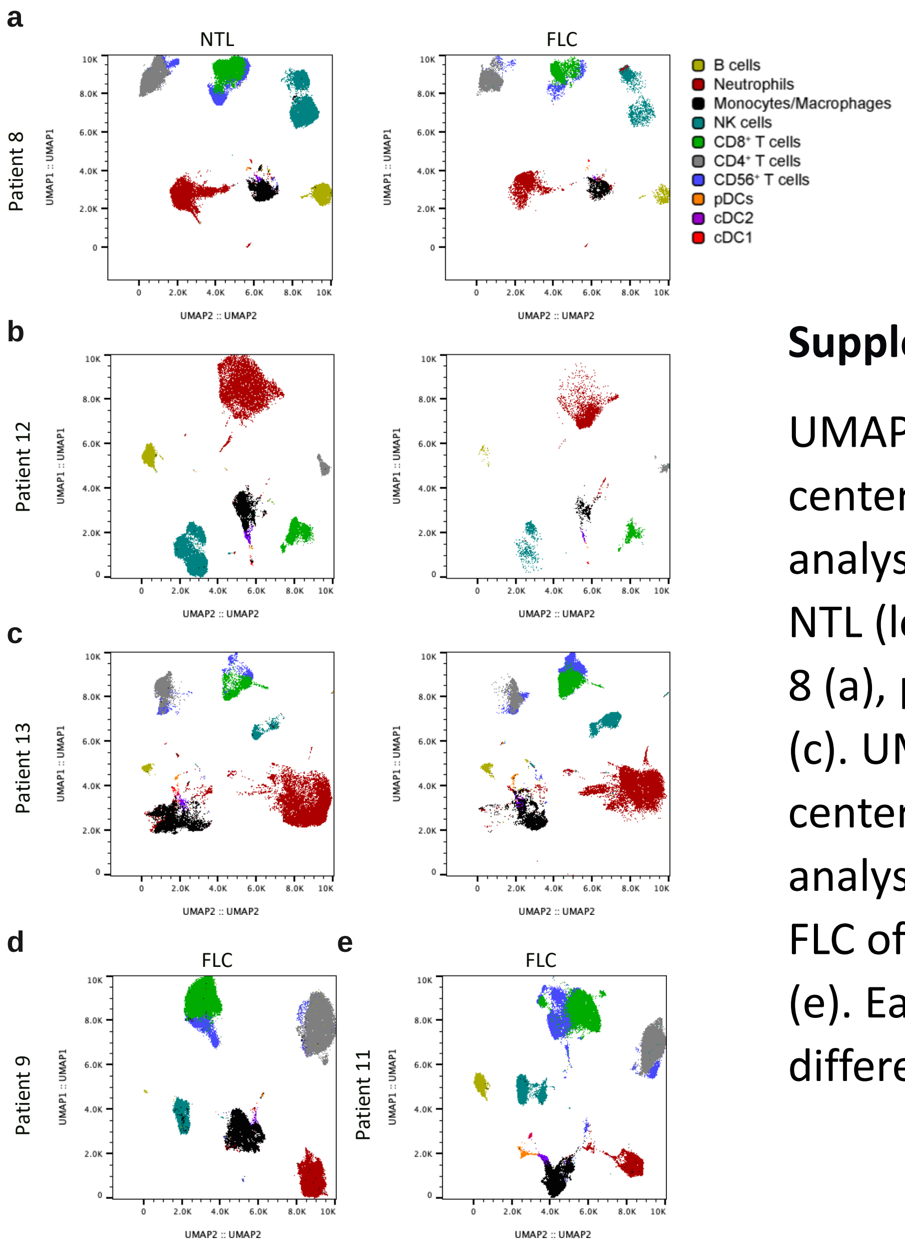

Supplementary figure S3.

UMAPs displaying myeloid-centered panel flow cytometry analysis of live CD45^{+} cells from NTL (left) and FLC (right) of patient 8 (a), patient 12 (b) and patient 13 (c). UMAPs displaying myeloid-centered panel flow cytometry analysis of live CD45^{+} cells from FLC of patient 9 (d) and patient 11 (e). Each color represents a different immune population.

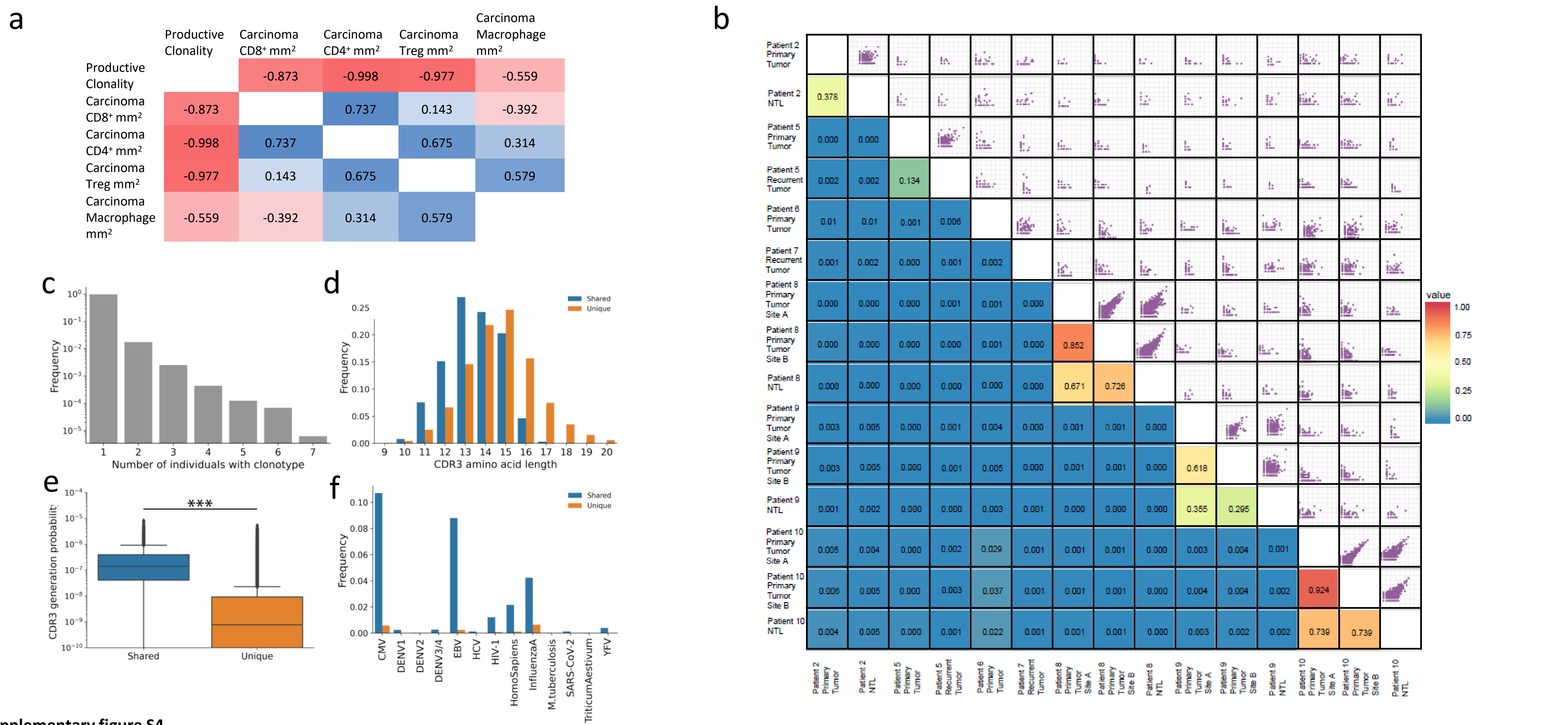

Supplementary figure S4.

(a) Correlation matrix comparing productive T cell clonality in the tumor as well as CD8+ T cell, CD4+ T cell, Regulatory T cell, and macrophage densities in the carcinoma compartment of FLC tumors

(b) Overlap of TCR amino acid sequences for FLC tumor and NTL samples displayed as an Morisita's overlap index (MOI) heat map and scatterplot matrix

(c) Distribution showing the frequency of T cell receptor CDR3β amino acid sequence clonotypes across individuals. (d) Distribution of CDR3 amino acid lengths demonstrates that clonotypes shared across at least two individuals are considerably shorter than those found in only a single individual ($p<0.001$ by Mann-Whitney U test). (e) Shared TCR sequences had significantly higher generation probabilities (***$p<0.001$ by Mann-Whitney U test) as calculated by OLGA[7] (f) Shared TCR sequences were significantly more likely to have been previously found to recognize common viral epitopes in VDJdb[8]. CMV- cytomegalovirus, DENV- Dengue virus, EBV- Epstein-Barr virus, HCV- Hepatitis C virus, HIV- human immunodeficiency virus, YFV- Yellow fever virus.

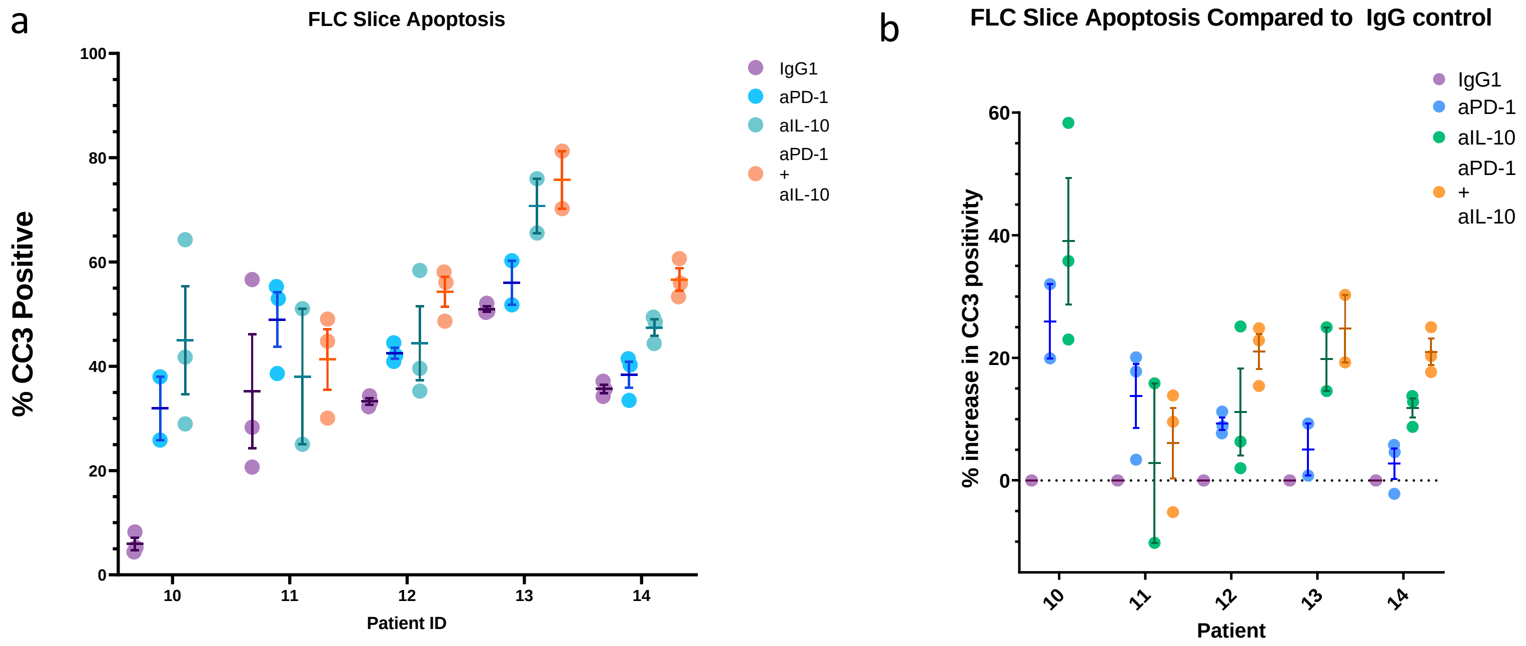

Supplementary figure S5.

Cleaved caspase 3 (cC3) after different treatment groups for individual patients with (a) percentage absolute values and (b) percentage increased using IgG control as baseline of zero

Position	Antibody	Clone / Host	Company / Item	Concentration	OPAL (1:100)
1	CK18	EP30 / Rabbit	Bio SB / BSB 6698	0.5 ug/ml	520
2	CD8	144B / Mouse	DAKO / M7103	0.05 ug/ml	540
3	CD68	PG-M1 / Mouse	DAKO / M0876	0.04 ug/ml	570
4	HLA-DR	EP96 / Rabbit	Bio SB / BSB 6797	0.13 ug/ml	620
5	CD4	EP204 / Rabbit	Epitomics / AC0173A	0.08 ug/ml	690
6	FoxP3	236A/E7 / Mouse	eBioscience / 14-4777-82	5 ug/ml	650

Supplemental Table 1

Multiplex immunohistochemistry (mIHC) primary antibodies

Marker	Fluorophore	Clone	Vendor	Catalog #	Host species	Species Reactivity
CX3CR1	BV421	2A9-1	BD Biosciences	565800	Rat	Human
CD16	BUV496	3G8	BD Biosciences	612944	Mouse	Human
CD1c	AF647	F10/21A3	BD Biosciences	565048	Mouse	Human
CD11c	AF700	B-ly6	BD Biosciences	561352	Mouse	Human
CD123	BV786	7G3	BD Biosciences	564196	Mouse	Human
CD4	PE-Cy7	SK3	BD Biosciences	557852	Mouse	Human
CD141	BV605	1A4	BD Biosciences	740421	Mouse	Human
HLA-DR	APC-H7	G46-6	BD Biosciences	561358	Mouse	Human
CD3	BUV661	UCHT1	BD Biosciences	612964	Mouse	Human
CD45	BUV805	HI30	BD Biosciences	612891	Mouse	Human
CD11b	BV750	ICRF44	BD Biosciences	747357	Mouse	Human
CD14	BV711	MφP9	BD Biosciences	563372	Mouse	Human
CD45	BUV563	HI30	BD Biosciences	748720	Mouse	Human
CD294 (CRTH2)	PE-CF594	BM16	BD Biosciences	563501	Rat	Human
CD16	BUV395	3G8	BD Biosciences	563785	Mouse	Human
CD3	BUV496	UCHT1	BD Biosciences	612940	Mouse	Human
CD14	PerCP-Cy 5.5	MφP9	BD Biosciences	562692	Mouse	Human
HLA-DR	BUV661	G46-6	BD Biosciences	612980	Mouse	Human
CD25	BUV563	2A3	BD Biosciences	612918	Mouse	Human
CD4	BUV496	SK3	BD Biosciences	612936	Mouse	Human
CD279 (PD-1)	BV786	EH12.1	BD Biosciences	563789	Mouse	Human
CD3	APC-H7	SK7	BD Biosciences	560176	Mouse	Human
CD8	BB660	RPA-T8	BD Horizon™ Customs		Mouse	Human
CD127	BB790	HIL-7R-M21	BD Horizon™ Customs		Mouse	Human
TCR γδ	PE-Cy5	B1	BD Horizon™ Customs		Mouse	Human
CD56	BB790	NCAM16.2	BD Horizon™ Customs		Mouse	Human
CD161	APC-Fire 750	HP-3G10	Biolegend	339944	Mouse	Human
CD197 (CCR7)	PE	G043H7	Biolegend	353204	Mouse	Human
CD45RA	BV570	HI100	Biolegend	304132	Mouse	Human
CD20	PerCP-Cy 5.5	2H7	Biolegend	302326	Mouse	Human
TCR Va7.2	BV785	3C10	Biolegend	351722	Mouse	Human
CD19	PerCP-Cy 5.5	SJ25C1	Biolegend	363016	Mouse	Human
CD335 (NKp46)	Biotin	9E2	Biolegend	331906	Mouse	Human
CD33	PerCP-Cy 5.5	WM53	Biolegend	303414	Mouse	Human
CD8a	BV605	RPA-T8	Biolegend	301040	Mouse	Human
CD152 (CTLA-4)	BV421	BNI3	Biolegend	369606	Mouse	Human
Streptavidin	PE		Biolegend	405204		
CD19	PE-Cy5.5	SJ25C1	Invitrogen (Thermofisher Science)	35-0198-42	Mouse	Human
FoxP3	PE-Cy5.5	PCH101	Invitrogen (Thermofisher Science)	35-4776-42	Rat	Human
CD117 (cKit)	PE-Cy5.5	104D2	Invitrogen (Thermofisher Science)	CD11718	Mouse	Human

Supplemental Table 2

Flow cytometry antibodies
